# Development of a Multiplex Real-Time PCR Assay for the Detection of Eight Pathogens Associated with Bovine Respiratory Disease Complex from Clinical Samples

**DOI:** 10.3390/microorganisms13071629

**Published:** 2025-07-10

**Authors:** Fuxing Hao, Chunhao Tao, Ruilong Xiao, Ying Huang, Weifeng Yuan, Zhen Wang, Hong Jia

**Affiliations:** 1 Jiangsu Agri-Animal Husbandry Vocational College, Taizhou 225300, China; vethfx@163.com; 2 Institute of Animal Sciences, Chinese Academy of Agricultural Sciences, Beijing 100193, China; chunhao_tao@163.com (C.T.); xiaoruilong1207@163.com (R.X.); hy811cysbm@163.com (Y.H.); yuanweifeng@caas.cn (W.Y.); wz2893963594@126.com (Z.W.)

**Keywords:** bovine respiratory disease complex (BRDC), multiplex qPCR, diagnosis, mixed infection

## Abstract

Bovine respiratory disease complex (BRDC) is one of the primary causes of morbidity, mortality, and economic loss in cattle worldwide. Accurate and rapid identification of causative pathogenic agents is essential for effective disease management and control. In this study, a novel multiplex fluorescence-based quantitative polymerase chain reaction (qPCR) assay was developed for the simultaneous detection of eight major pathogens associated with BRDC. The targeted pathogens included the following: bovine viral diarrhea virus (BVDV), bovine parainfluenza virus type 3 (BPIV3), bovine respiratory syncytial virus (BRSV), bovine coronavirus (BcoV), *Mycoplasma bovis* (M.bovis), *Pasteurella multocida* (PM), *Mannheimia haemolytica* (MH), and infectious bovine rhinotracheitis virus (IBRV). The assay was rigorously optimized to ensure high specificity with no cross-reactivity among targets. The limit of detection (LOD) was determined to be as low as 5 copies per reaction for all target pathogens. The coefficient of variation (CVs) for both intra-assay and inter-assay measurements were consistently below 2%, demonstrating excellent reproducibility. To validate the clinical utility of the assay, a total of 1012 field samples were tested, including 504 nasal swabs from Farm A and 508 from Farm B in Jiangsu Province. BVDV, BcoV, PM, and MH were detected from Farm A, with a BVDV-positive rate of 21.63% (109/504), BcoV-positive rate of 26.79% (135/504), PM-positive rate of 28.77% (145/504), and MH-positive rate of 15.08% (76/504). Also, BcoV, PM, MH, and IBRV were detected from Farm B, with a BcoV-positive rate of 2.36% (12/508), PM-positive rate of 1.38% (7/508), MH-positive rate of 14.76% (75/508), and IBRV-positive rate of 5.51% (28/508). Notably, a significant proportion of samples showed evidence of mixed infections, underscoring the complexity of BRDC etiology and the importance of a multiplex diagnostic approach. In conclusion, the developed multiplex qPCR assay provides a reliable, rapid, and cost-effective tool for simultaneous detection of multiple BRDC-associated pathogens, which will hold great promise for enhancing disease surveillance, early diagnosis, and targeted intervention strategies, ultimately contributing to improved BRDC management and cattle health outcomes.

## 1. Introduction

Bovine respiratory disease complex (BRDC) is a multifactorial disease that significantly impacts cattle health and productivity worldwide. BRDC represents a multifactorial syndrome caused by multiple pathogens [1], including viruses such as the bovine viral diarrhea virus (BVDV) [2], infectious bovine rhinotracheitis virus (IBRV) [3], bovine parainfluenza virus type 3 (BPIV3) [4], bovine respiratory syncytial virus (BRSV) [5], and bovine coronavirus (BcoV) [6], as well as bacteria such as *Mannheimia haemolytica* (MH) [7], *Mycoplasma bovis* (M.bovis) [8], and *Pasteurella multocida* (PM) [9].

The pathogenesis of BRDC is complex and involves an intricate interplay between primary viral infections and secondary bacterial colonization [10]. Viruses such as BVDV, BPIV3, BRSV, IBRV, and BcoV often initiate the disease process by damaging the respiratory epithelium and impairing host immune defenses [11]. This disruption creates a conducive environment for opportunistic bacteria like M.bovis, PM, and MH to invade and exacerbate the condition [12,13]. The synergistic effects among these pathogens result in severe respiratory distress, making BRDC a formidable challenge for veterinarians and livestock producers.

BRDC predominantly affects young calves and feedlot cattle, leading to high morbidity and mortality rates, reduced growth performance, increased treatment costs, and substantial economic losses [14]. Globally, similar patterns are observed, particularly in regions with intensive cattle farming systems where animals are exposed to high-density housing, transportation stress, and fluctuating environmental conditions. In North America, BRDC is the leading cause of morbidity and mortality in feedlot cattle, with reported incidence rates ranging from 15% to 40% in high-risk populations [15]. Similarly, in Europe, BRDC outbreaks are common during cold seasons and in poorly ventilated housing systems. Studies have shown that up to 30% of cattle in European herds may be affected annually [16]. In developing regions, such as parts of Africa and Asia, BRDC poses an even greater challenge due to limited access to vaccines, inadequate biosecurity measures, and reliance on traditional farming practices. For instance, in Inner Mongolia, where smallholder farmers dominate the livestock sector, BRDC outbreaks due to poor nutrition and harsh environmental conditions [17]. The coexistence of these multiple pathogens in affected cattle complicates diagnosis and treatment. Mixed infections are frequently observed in BRDC cases, with multiple pathogens detected simultaneously in clinical samples [10]. This complexity underscores the need for diagnostic tools capable of identifying all potential causative agents in single assay, rather than relying on sequential testing methods that may delay accurate diagnosis and appropriate intervention.

Despite decades of research into BRDC, diagnosing the disease remains a significant challenge due to its multifactorial nature and the limitations of existing diagnostic methods [18]. Traditional approaches, such as bacterial culture, serological assays, and single-plex PCR tests, have several drawbacks that hinder their effectiveness in clinical settings. Furthermore, many conventional methods also fail to reliably detect mixed infections, which are a hallmark of BRDC and contribute significantly to disease severity [19]. Recent advancements in molecular diagnostic technologies, such as next-generation sequencing (NGS) and metagenomic analysis, have provided promising tools for pathogen identification and characterization [20]. However, these technologies are expensive, require sophisticated equipment and expertise, and are not readily accessible for routine use in most veterinary laboratories. Therefore, there remains an urgent need for a rapid, cost-effective, and multiplex diagnostic tool capable of simultaneously detecting and differentiating the major pathogens associated with BRDC.

Multiplex PCR is a technique that allows for simultaneous amplification of multiple target sequences within a single reaction system. It offers advantages such as simplicity, high sensitivity, and strong specificity and is widely used in the detection of pathogenic microorganisms [21,22]. To address the aforementioned challenges, a novel multiplex qPCR assay capable of detecting and differentiating eight major BRDC-associated pathogens—BVDV, BPIV3, BRSV, BcoV, M.bovis, PM, MH, and IBRV—was developed. A set of primers and probes targeting conserved genomic regions of these eight pathogens was designed and optimized. The assay was divided into two reaction tubes to maximize efficiency while minimizing the risk of cross-reactivity. The analytical performance of the assay was evaluated in terms of sensitivity, specificity, reproducibility, and its ability to detect mixed infections. The clinical utility of the assay was further assessed using field samples from cattle. In summary, this study aimed to provide a robust, rapid, and cost-effective diagnostic solution that could enhance disease surveillance, facilitate timely interventions, and ultimately improve BRDC management strategies worldwide.

## 2. Materials and Methods

### 2.1. Ethical Approval and Sample Collection

All procedures involving the collection and handling of animal samples were conducted in strict accordance with the guidelines established by the Institutional Animal Care and Use Committee (IACUC) of Jiangsu Agri-animal Husbandry Vocational College. Ethical approval was obtained prior to the initiation of the study (Approval Number: Jsahvc-2025-73). A total of 1012 field samples were collected from 2-year-old Simmental cattle from two commercial cattle farms in Jiangsu Province, following informed consent from farm owners. The animals were housed under intensive housing systems and had been vaccinated against lumpy skin disease. Nasal swabs were collected as primary sample types due to their relevance in detecting respiratory pathogens. All samples were immediately placed in sterile containers containing 0.9% sodium chloride solution and transported on ice to the laboratory for processing within 24 h.

### 2.2. Primer and Probe Design

The nucleotide sequences of conserved genes or virulence genes of eight BRDC-associated pathogens that have been prevalent in China in recent years were retrieved from GenBank (NCBI). Multiple sequence alignments were conducted using the Clustal W algorithm implemented in MEGA 11 software to identify conserved genomic regions among different strains of each target pathogen. Subsequently, regions that are conserved among different strains of the same pathogen but specific relative to other common bovine pathogens were selected for designing specific primers and probes to ensure broad detection capability while maintaining specificity relative to other common bovine pathogens. To minimize cross-reactivity, all primers and probes were subjected to in silico specificity testing using Primer-BLAST (https://www.ncbi.nlm.nih.gov/tools/primer-blast/ accessed on 15 March 2024). Probes were labeled with distinct fluorophores, including FAM, VIC, ROX, and Cy5, enabling simultaneous detection of multiple targets. Each probe was quenched with a corresponding dark quencher (BHQ2 or MGB) to reduce background fluorescence. The above primers and probes are shown in Table 1 and were synthesized by Shanghai Jierui Biotechnology Co., Ltd. (Shanghai, China).

### 2.3. Design Multiplex qPCR Assay

Based on the eight designed primer–probe sets, the corresponding target fragments were synthesized and cloned into the pUC57 plasmid vector to generate eight standard plasmids, which served as positive controls and were used for assay validation. These plasmids were constructed by Nanjing GenScript Biotechnology Co., Ltd. (Nanjing, China). In order to obtain RNA transcripts, four of the aforementioned standard plasmids were transcribed in vitro using the HiScribe^®^ T7 High Yield RNA Synthesis Kit (New England Biolabs, Ipswich, MA, USA). Then the transcribed RNAs were column-purified with the RNA Clean & Concentrator Kit (Zymo Research, Irvine, CA, USA). Finally, the purified RNA transcripts were quantified using a NanoDrop spectrophotometer (Thermo Fisher Scientific, Waltham, MA, USA), and their concentrations were calculated accordingly.

To optimize amplification efficiency and minimize cross-reactivity among targets, the eight primer–probe sets were divided into two separate reaction panels. Four primer–probe sets targeting BVDV, BPIV3, BRSV, and BcoV were combined into Primer Pool A, while the remaining four sets targeting M.bovis, PM, MH, and IBRV were grouped into Primer Pool B.

Four standard plasmids (10^5^ copies/μL) containing the target DNA sequences from Primer Pool B, and RNA transcripts (10^5^ copies/μL) corresponding to the four RNA targets from Primer Pool A, were used as templates to evaluate the performance of each primer–probe set. In addition, nucleic acids extracted from other bovine infectious pathogens routinely preserved in our laboratory were included as negative controls to assess assay specificity. Each primer–probe set in Primer Pool A was tested individually using the Hifair^®^ III One Step RT-qPCR Probe Kit (Yeasen Biotechnology Co., Ltd., Shanghai, China), while those in Primer Pool B were tested using the Taq Pro HS U^+^ Probe Master Mix (Vazyme International LLC, Nanjing, China), both following the respective manufacturers’ instructions. The qPCR amplification was carried out using the SLAN-96S fluorescence quantitative PCR instrument (Shanghai Hongshi Medical Technology Co., Ltd., Shanghai, China). Fluorescence signals were detected using the FAM, VIC, ROX, and Cy5 fluorescence channels during the extension phase.

### 2.4. Optimization of Reaction Conditions for Multiplex Fluorescence Quantitative PCR

#### 2.4.1. Optimization of Primer and Probe Concentrations in Two Reaction Pools

To optimize the amplification efficiency and specificity of the multiplex qPCR assay, the concentrations of primers and probes within each primer pool were systematically evaluated. For each target gene, a series of working concentrations of forward and reverse primers (100 nM, 150 nM, 200 nM, 250 nM, and 300 nM) and probes (50 nM, 100 nM, 150 nM, 200 nM, and 250 nM) were tested. While varying one primer–probe set, the concentrations of the other primer and probe set in the same pool were kept constant (200 nmol/L) to avoid interference. The plasmids or RNA transcripts containing the corresponding target fragments were used as templates for amplification. Each concentration was tested in triplicate using either the Hifair^®^ III One Step RT-qPCR Probe Kit (for RNA templates) or the Taq Pro HS U^+^ Probe Master Mix (for DNA templates), respectively, and fluorescence signals were detected using the SLAN-96S Real-Time PCR System. The detailed composition of the reaction systems is presented in Table 2. The reaction conditions were based on the manufacturers’ recommended protocols. Data analysis was performed using the SLAN software (Version 8.2.2) (Shanghai Hongshi Medical Technology Co., Ltd., Shanghai, China), and amplification curves and Ct values were automatically generated by the system. The optimal primer and probe concentrations were determined based on Ct values and signal intensity.

#### 2.4.2. Determination of Optimal Annealing Temperature

A standard plasmid or an RNA transcript containing the target fragment was used as the template at a concentration of 10^5^ copies/μL. qPCR was performed using the SLAN-96S instrument. Three technical replicates were tested for each condition to determine the optimal annealing temperature for both Primer Pool A and Primer Pool B. The reaction system and cycling conditions were consistent with those described above, except for the annealing temperatures, which were tested in 2 °C increments ranging from 56 °C to 64 °C to identify the optimal amplification conditions for each primer pool.

#### 2.4.3. Development of Standard Curves for Eight Target Pathogens

Four standard plasmids and four RNA transcripts with known copy numbers were subjected to 10-fold serial dilutions ranging from 10^2^ to 10^7^ copies/μL. qPCR was then performed for each dilution using the multiplex qPCR assay developed in this study and ran on the SLAN-96S Real-Time PCR System. Each concentration was tested in triplicate to ensure assay repeatability and linearity. The resulting Ct values were plotted against the logarithm of the template copy numbers using GraphPad Prism 8 software (GraphPad Software, San Diego, CA, USA) to generate standard curves for each target pathogen. The linear regression equation and coefficient of determination (R^2^) were calculated to evaluate the correlation between Ct values and template concentrations.

### 2.5. Analytical Performance Evaluation

#### 2.5.1. Specificity Testing

To evaluate the analytical specificity of the multiplex qPCR assay, each primer–probe set was tested against a panel of nucleic acids derived from non-target pathogens and host genomic DNA extracted from bovine blood samples. The absence of cross-reactivity was confirmed through analysis of the amplification curves from each reaction. Additionally, standard plasmids (for Primer Pool B) or RNA transcripts (for Primer Pool A) representing the conserved target regions of each pathogen were used as positive controls to ensure accurate and specific detection.

#### 2.5.2. Sensitivity Testing

The limit of detection (LOD) for each of the eight target pathogens was determined using serial dilutions of plasmid DNA or in vitro-transcribed RNA. Mixtures of plasmids (Primer Pool B) or RNA transcripts (Primer Pool A), representing all four respective targets in each panel, were prepared at final concentrations of 10, 5, 1, and 0.5 copies/μL. Each concentration was tested in 8 technical replicates to assess the detection capability of the assay. The LOD was defined as the lowest concentration at which 95% of replicates yielded a positive amplification signal. This evaluation confirmed the assay’s ability to reliably detect pathogens at low concentrations in clinical samples.

#### 2.5.3. Reproducibility Testing

Reproducibility was assessed by evaluating both intra-assay and inter-assay variability based on the coefficients of variation (CVs) of Ct values. Intra-assay variability was assessed by running 16 replicates of the same sample within a single experiment, while inter-assay variability was calculated from results obtained across three independent runs conducted on different days. Templates (including standard plasmids and RNA transcripts) were diluted to concentrations of 10^5^ copies/μL and 10^2^ copies/μL, respectively. Ct values were recorded for each replicate following amplification, and the mean Ct, standard deviation (SD), and CVs were computed using Excel. A CV below 5% was considered an acceptable threshold for reproducibility, for both intra-assay and inter-assay variability [23].

### 2.6. Analysis of Real-World Clinical Samples

Clinical validation was performed using field samples collected from cattle exhibiting clinical signs consistent with bovine respiratory disease complex (BRDC). A total of 1012 field samples were tested, including 504 nasal swabs from Farm A and 508 from Farm B in Jiangsu Province. DNA/RNA was extracted from each sample as previously described [22]. Then the purified nucleic acids were subjected to multiplex qPCR using the assay developed in this study on the SLAN-96S Real-Time PCR System. The Ct values obtained from all positive amplification reactions were recorded. To assess the accuracy and reliability of the developed multiplex qPCR assay in clinical sample detection, a parallel validation experiment was conducted using national reference methods or previously published TaqMan qPCR assays capable of detecting the same eight BRDC-associated pathogens [24,25,26,27]. In detail, all samples that tested positive with the developed assay, along with an additional 100 negative samples, were independently analyzed using these reference methods according to their respective protocols.

### 2.7. Statistical Analysis

All data management and graphical presentations were carried out using GraphPad Prism 8 software (GraphPad Software, San Diego, CA, USA). For the evaluation of sample size adequacy, a power analysis was conducted using the pwr package (version 1.3-0) in R (version 4.2.2). The two-sample proportion test was applied, assuming a minimum Cohen’s h effect size of 0.2 (small effect), an α level of 0.01, and desired power of 0.90. These parameters ensured a rigorous assessment of the minimum sample size to detect true differences in pathogen detection rates between the two farms.

## 3. Results

### 3.1. Establishment of Multiplex qPCR Method

#### 3.1.1. Validation of Primer Pool Performance

To verify the performance of the designed primer–probe sets, four pairs targeting BVDV, BPIV3, BRSV, and BcoV were combined into Primer Pool A, while the remaining four targeting M.bovis, PM, MH, and IBRV were grouped into Primer Pool B. Since the pathogens in Primer Pool A are RNA viruses, whereas those in Primer Pool B are DNA viruses, the use of corresponding nucleic acid types during assay development was considered essential to more accurately reflect their potential performance in clinical sample detection. Therefore, after constructing standard plasmids for all eight targets, the four plasmids corresponding to the pathogens in Primer Pool A were subjected to in vitro transcription to generate RNA transcripts. Accordingly, the assay for Primer Pool A was developed using these four RNA transcripts as templates, whereas that for Primer Pool B was developed using the respective plasmid DNAs. Specifically, each primer–probe set was tested using standard plasmids (Primer Pool B) or corresponding RNA transcripts (Primer Pool A) containing the target gene sequences, diluted to a concentration of 10^5^ copies/μL. Amplification curves confirmed that each primer–probe set specifically detected its intended target without cross-reactivity. The qPCR results demonstrated clear amplification signals for all target pathogens in both primer pools, with Ct values ranging from 19 to 22 when the corresponding nucleic acid templates were present. These findings indicate that both Primer Pool A and Primer Pool B exhibit high specificity and consistent amplification efficiency. The detailed results are shown in Table 3.

#### 3.1.2. Optimization of Primer and Probe Concentrations in Primer Pools

**Figure 1:**
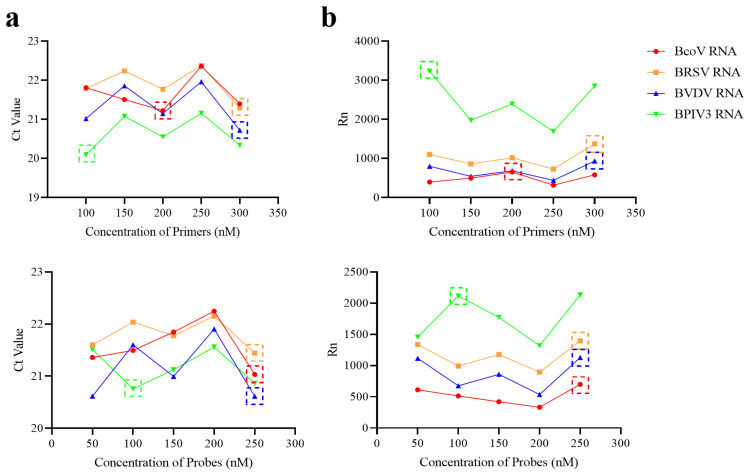
Optimization results of primer and probe concentration in Primer Pool A. (**a**) The mean Ct value for each concentration of primers and probes in Primer Pool A. (**b**) The mean Rn value for each concentration of primers and probes in Primer Pool A. The dotted boxes indicate the optimal concentration selected.

**Figure 2:**
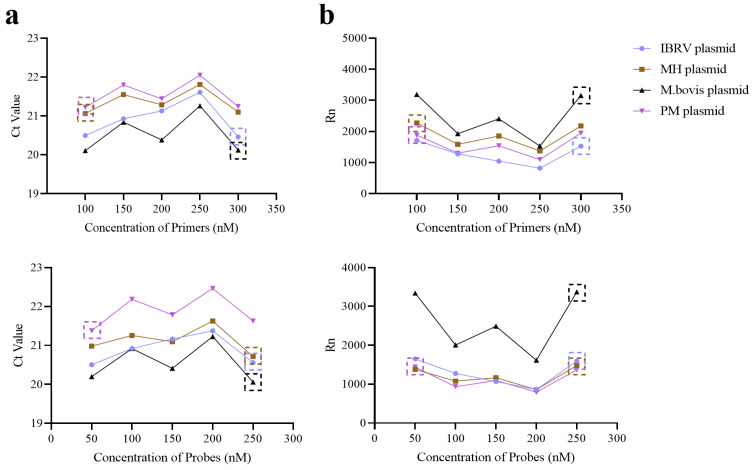
Optimization results of primer and probe concentration in Primer Pool B. (**a**) The mean Ct value for each concentration of primers and probes in Primer Pool B. (**b**) The mean Rn value for each concentration of primers and probes in Primer Pool B. The dotted boxes indicate the optimal concentration selected.

To determine the optimal working concentrations for each primer–probe set, a series of gradient dilutions were tested. Forward and reverse primers targeting the conserved regions of each pathogen were evaluated at final concentrations of 100 nM, 150 nM, 200 nM, 250 nM, and 300 nM. Probes were prepared at working concentrations of 50 nM, 100 nM, 150 nM, 200 nM, and 250 nM. During optimization, all other primer and probe concentrations within the same pool were held constant (200 nmol/L) to avoid interference effects. A plasmid or RNA transcript containing the target fragment was used as a template for amplification under each condition. The performance of each concentration combination was evaluated based on mean Ct values and absolute fluorescence signal intensities (Rn values). For Primer Pool A, mean Ct values are shown in Figure 1a, and the Rn values are presented in Figure 1b. Similarly, results for Primer Pool B are displayed in Figure 2. The optimal concentration was defined as the one yielding the lowest Ct value, indicating the highest amplification efficiency. In cases where multiple concentrations produced similar Ct values, the concentration associated with the highest Rn value—indicating better detection sensitivity—was selected. Based on this systematic evaluation, the final optimized concentrations for all primers and probes were determined and used in subsequent validation experiments (Figure 1 and Figure 2).

#### 3.1.3. Optimal Annealing Temperature

**Figure 3:**
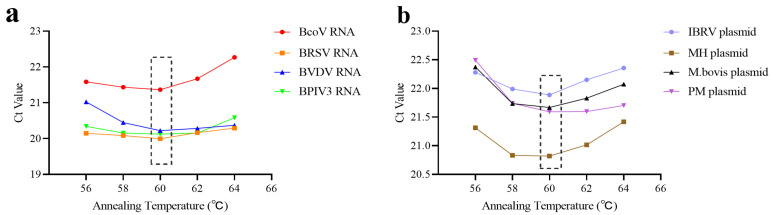
Results of annealing temperature optimization. (**a**) The mean Ct value for each annealing temperature of Primer Pool A. (**b**) The mean Ct value for each annealing temperature of Primer Pool B. The dotted boxes indicate the optimal annealing temperature selected.

Following the determination of the optimal primer and probe concentrations, reaction conditions were further optimized by evaluating a range of annealing temperatures. Temperatures included 56 °C, 58 °C, 60 °C, 62 °C, and 64 °C were tested. A standard plasmid or RNA transcript containing the target fragment was diluted to a concentration of 10^5^ copies/μL and used as the template. The qPCR assay was performed using both Primer Pool A and Primer Pool B under each temperature condition. The optimal annealing temperature was defined as the one yielding the lowest Ct value, indicating the highest amplification efficiency. The reaction temperature of 60 °C yielded the most favorable amplification performance for both primer pools, as evidenced by significantly lower Ct values across all target pathogens, demonstrating that an annealing temperature of 60 °C is optimal for the developed multiplex qPCR assay (Figure 3).

#### 3.1.4. Construction of Standard Curves

**Figure 4:**
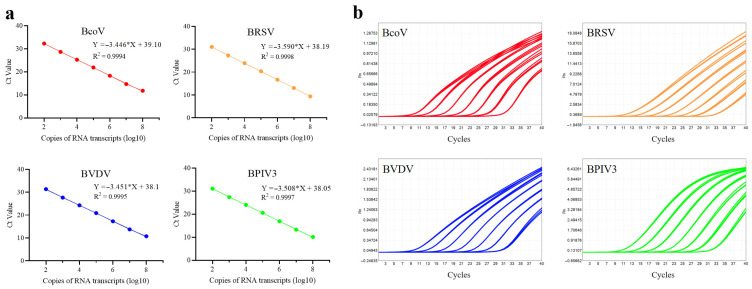
Constructions of standard curves of Primer Pool A. (**a**) Standard curves generated by Primer Pool A for four target RNA transcripts. (**b**) Amplification curves corresponding to the standard curves for four target RNA transcripts. Each test was conducted with three replicates. The asterisk (*) represents the multiplication sign in linear equations.

**Figure 5:**
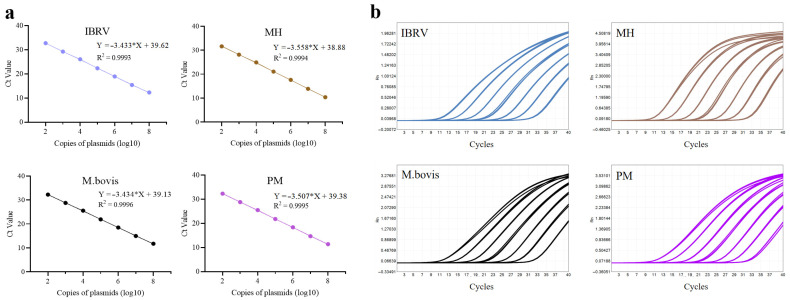
Constructions of standard curves of Primer Pool B. (**a**) Standard curves generated by Primer Pool B for four target plasmids. (**b**) Amplification curves corresponding to the standard curves for four target plasmids. Each test was conducted with three replicates. The asterisk (*) represents the multiplication sign in linear equations.

Standard curves were constructed under optimized reaction conditions to assess the amplification efficiency and linearity of the multiplex qPCR assay. Serial 10-fold dilutions of plasmid DNA or transcribed RNA containing target sequences were prepared, ranging from 10^8^ to 10^2^ copies/µL. Following the qPCR run, standard curves were constructed by plotting the Ct values obtained from qPCR amplification on the y-axis against the logarithm of the template copy numbers on the x-axis. The standard curves for Primer Pool A are shown in Figure 4, and those for Primer Pool B are shown in Figure 5. The slopes of the standard curves for the targets in Primer Pool A of BVDV, BPIV3, BRSV, and BcoV RNA transcripts were −3.451 (amplification efficiency: 94.88%), −3.508 (92.78%), −3.590 (89.91%), and −3.446 (95.07%), respectively. The slopes of the standard curves for the targets in Primer Pool B of M.bovis, PM, MH, and IBRV plasmids were −3.434 (95.53%), −3.507 (92.82%), −3.558 (91.01%), and −3.433 (95.56%), respectively. The R^2^ values for all targets in Primer Pool A and Primer Pool B exceeded 0.999, demonstrating excellent linearity and efficient amplification. These results confirm that both primer pools exhibit excellent amplification performance, with high sensitivity and reproducibility over a wide dynamic range.

### 3.2. Specificity Tests

**Figure 6:**
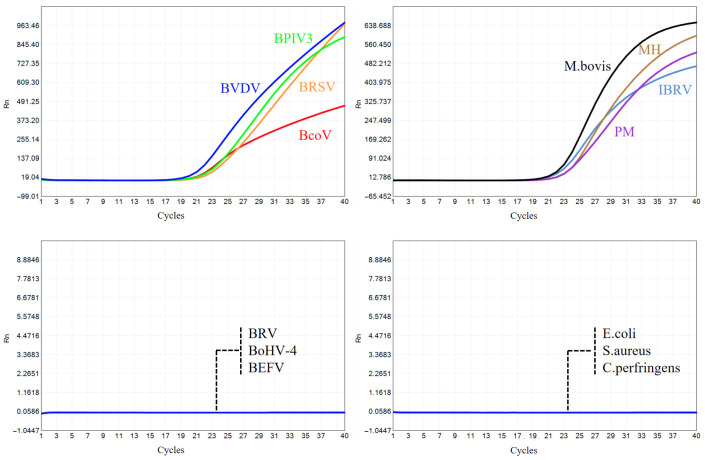
Amplification curves of specificity tests.

The specificity of the developed multiplex qPCR assay was evaluated using standard plasmids or RNA transcripts containing target gene sequences. In addition, the assay was tested against nucleic acids from non-target bovine pathogens stored in our laboratory, including bovine rotavirus (BRV), bovine epizootic fever virus (BEFV), bovine herpesvirus 4 (BoHV-4), *Escherichia coli* (*E. coli*), *Staphylococcus aureus* (*S. aureus*), and *Clostridium perfringens* (*C. perfringens*). The amplification curves demonstrated that the established method exhibits high specificity to the target nucleic acids, with no cross-reaction to non-target pathogens or host DNA extracted from bovine blood samples (Figure 6).

### 3.3. Sensitivity Tests

**Figure 7:**
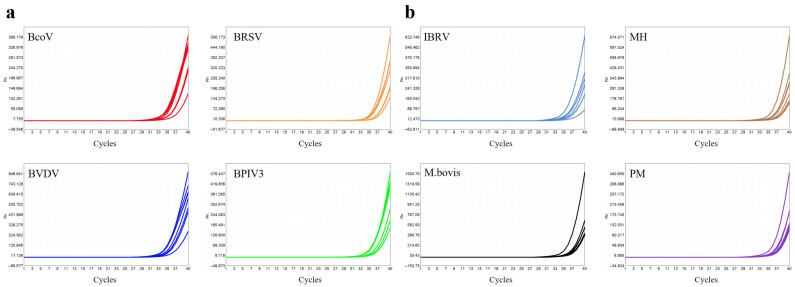
Amplification curves of sensitivity tests. (**a**) Amplification curves of Primer Pool A for four target RNA transcripts (8 replicates each) at a concentration of 5 copies/μL. (**b**) Amplification curves of Primer Pool B for four target plasmids (8 replicates each) at a concentration of 5 copies/μL.

Analytical sensitivity was assessed by determining the limit of detection (LOD) for each target using serial dilutions of plasmid DNA or in vitro-transcribed RNA, ranging from 10 to 0.5 copies/µL. At 5 copies/µL, ≥95% of replicates tested positive (Table 4), indicating an LOD of 5 copies/μL across all eight targets. Representative amplification curves are shown in Figure 7, confirming the assay’s ability to detect low-abundance pathogens typically found in clinical specimens.

### 3.4. Reproducibility Tests

The repeatability and consistency of the multiplex qPCR assay were evaluated through intra-assay and inter-assay assessments. Standard plasmids or RNA transcripts at 10^5^ and 10^2^ copies/μL were used as templates, with 16 replicates tested per run to assess intra-assay variability. Inter-assay variability was determined across three independent runs conducted on separate days. As presented in Table 5, mean Ct values derived from 16 replicates per target showed minimal variation, with standard deviations ranging from 0.04 to 0.39. Intra-assay and inter-assay coefficients of variation (CVs) remained below 2%, confirming the high precision and reproducibility of the assay. These results demonstrate their suitability for accurate and reliable detection and quantification of BRDC-associated pathogens in routine diagnostics.

### 3.5. Clinical Sample Testing

**Figure 8:**
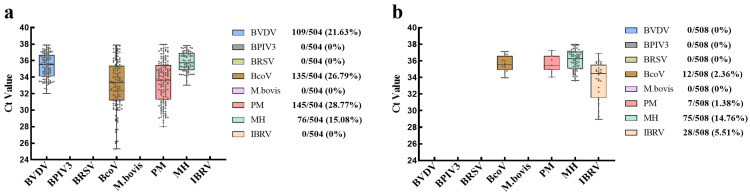
Results of clinical sample testing. (**a**) Detection results of eight pathogens and associated Ct value distributions in Farm A (*n* = 504). (**b**) Detection results of eight pathogens and associated Ct value distributions in Farm B (*n* = 508). Each dot in the figure represents a positive sample, with its position on the y-axis indicating the corresponding Ct value.

A total of 1012 nasal swab samples were collected from cattle displaying clinical signs consistent with BRDC across two commercial farms in Jiangsu Province. All samples were tested using the developed multiplex qPCR assay. In Farm A, BVDV, BcoV, PM, and MH were detected at positive rates of 21.63% (109/504), 26.79% (135/504), 28.77% (145/504), and 15.08% (76/504), respectively (Figure 8a). In Farm B, BcoV, PM, MH, and IBRV were identified at positive rates of 2.36% (12/508), 1.38% (7/508), 14.76% (75/508), and 5.51% (28/508), respectively (Figure 8b). BVDV was detected exclusively in Farm A, while IBRV was identified only in Farm B. BcoV, PM, and MH were detected in both farms, suggesting that these pathogens are more widely distributed across different farm settings. While both farms harbored four BRDC-associated pathogens, the overall prevalence in Farm A was significantly higher than in Farm B (Figure 8), underscoring the assay’s sensitivity and applicability in field diagnostics. The above results indicate that there are potential regional differences in pathogen prevalence within BRDC-affected cattle populations, highlighting the need for tailored disease control strategies.

Notably, a significant proportion of samples exhibited evidence of mixed infections. Among all detected samples from Farm A, 88 animals were infected with a single pathogen, while 101 were co-infected with two pathogens, 37 with three pathogens, and 16 with all four detected pathogens (Table 6). Detailed analysis of co-infection patterns in Farm A revealed that BcoV + PM was the most common combination (33 cases), followed by BVDV + PM (24 cases) and BVDV + BcoV (22 cases). Among triple infections, BcoV + PM + MH was relatively common (18 cases), and there were also a notable number of quadruple infections (16 cases). Compared to Farm A, the overall prevalence of most pathogens was lower in Farm B, except for MH (59 cases). Nevertheless, a notable number of co-infections were still detected, particularly IBRV + MH (15 cases) (Table 6). No quadruple infections were observed in Farm B. These findings underscore the complexity of BRDC etiology, reinforcing the multifactorial nature of the disease and highlighting the importance of multiplex detection for accurate diagnosis.

To validate the accuracy of clinical sample detection, a parallel comparison was conducted using national reference methods or previously published TaqMan qPCR assays capable of detecting the same eight BRDC-associated pathogens (Table 7). All samples that tested positive with the developed assay, along with an additional 100 negative samples, were retested using these reference methods. Kappa analysis of five detectable pathogens revealed specificity ranging from 96.00% to 100.00%, sensitivity from 89.79% to 97.37%, and overall agreement between methods ranging from 92.71% to 99.22% (Table 7). These findings demonstrate the high accuracy and reliability of the developed multiplex qPCR assay for clinical detection of BRDC-associated pathogens.

## 4. Discussion

Bovine respiratory disease complex (BRDC) is a major multifactorial disease responsible for considerable economic losses in cattle worldwide, primarily due to increased morbidity, mortality, and impaired growth performance [28]. It is commonly associated with co-infections involving both viral and bacterial agents, which often act synergistically to increase disease severity [10]. Current diagnostic tools, including bacterial culture, serology, and single-plex PCR, are constrained by their inability to simultaneously detect multiple pathogens and their limited utility for rapid decision-making. To address these limitations, we developed a two-tube multiplex qPCR assay for the simultaneous detection of eight key BRDC-associated pathogens: BVDV, BPIV3, BRSV, BcoV, M.bovis, PM, MH, and IBRV. This assay provides a rapid, sensitive, and economical platform for comprehensive diagnostics in veterinary practice.

In this study, four RNA viruses and four DNA pathogens (including bacteria) were selected. As RNA targets require an additional reverse transcription step and should be evaluated using RNA transcripts rather than plasmid DNA to better reflect their behavior in clinical samples, the corresponding primer–probe sets were assigned to Primer Pool A. In contrast, DNA targets do not require reverse transcription and were therefore grouped into Primer Pool B. For Primer Pool A, a one-step RT-qPCR protocol was employed, which integrates reverse transcription into the qPCR reaction without opening the tube, thereby reducing handling time, minimizing cross-contamination risk, and improving workflow efficiency. For Primer Pool B, a standard qPCR kit without reverse transcriptase was used, which lowered assay costs and ensured accurate representation of performance for DNA pathogen detection in clinical settings.

The multiplex qPCR assay developed in this study provides significant improvements over conventional diagnostic approaches. Its advantages include high analytical sensitivity and specificity, rapid processing time, and the capacity for simultaneous detection of multiple pathogens—all critical features for managing the complex etiology of BRDC. The assay demonstrated a limit of detection (LOD) as low as five copies/μL, enabling reliable pathogen identification even at very low concentrations. Such sensitivity is particularly valuable for early diagnosis, when pathogen loads may be insufficient for detection by less sensitive techniques [29]. Notably, BVDV, a highly contagious agent associated with immunosuppression, was consistently detected at five copies/μL, allowing for early intervention before infected animals contribute to viral spread [30].

The assay also demonstrated robust specificity through comprehensive in silico and experimental validation. Target-specific primers and probes were designed against conserved genomic regions unique to each pathogen, effectively eliminating cross-reactivity. Multiplex detection was achieved using spectrally distinct fluorophores (FAM, VIC, ROX, and Cy5), enabling clear discrimination of co-infecting agents without signal overlap. Notably, mixed infections were detected in approximately 20% of clinical samples, consistent with prior studies indicating the substantial contribution of co-infections to BRDC pathogenesis [8]. One representative case involved a nasal swab from a feedlot calf that tested positive for both BCoV and IBRV by the developed assay but was negative for IBRV via viral isolation [8]. This discrepancy underscores the superior performance of the assay in detecting pathogens that are challenging to isolate using conventional techniques [31].

The division of eight targets into two reaction tubes enables efficient use of reagents while preserving high throughput. This design significantly lowers operational costs and streamlines workflow, enhancing its suitability for use in laboratories with limited resources [32]. Compared with traditional diagnostic methods such as bacterial culture, serology, and single-plex PCR, the multiplex qPCR assay offers a more efficient and scalable approach. Bacterial culture, while considered the gold standard for identifying bacterial agents, is slow and limited in its ability to detect viruses. Serological assays are not ideal for early diagnosis and cannot differentiate between current and prior infections. Although single-plex PCR is highly sensitive and specific, it is impractical for routine detection of multiple pathogens due to its labor-intensive nature [33]. Advanced techniques such as next generation sequencing (NGS) offer broad pathogen discovery capabilities but remain cost-prohibitive and technically complex for routine diagnostics in most veterinary settings [34].

To ensure the adequacy of the sample size used in this study, a power analysis was also conducted based on a significance level (α) of 0.01, a Cohen’s h effect size of at least 0.2 (representing a small effect), and a target statistical power of 0.90. The analysis indicated a minimum required total sample size of 744 (at least 372 from each farm). Given that a total of 1012 field samples were collected (approximately 500 from each of the two farms), the actual sample size used in this study was more than sufficient to detect meaningful differences in pathogen prevalence between the two farms. This further supports the robustness and reliability of the diagnostic performance of the developed multiplex qPCR assay.

The development of this multiplex qPCR assay holds considerable promise for improving BRDC management in both clinical and epidemiological contexts. Rapid and precise identification of causative agents allows for targeted therapeutic and preventive strategies, including judicious use of antimicrobials and customized vaccination protocols. Accurate pathogen profiling helps minimize inappropriate antibiotic use, thereby contributing to the mitigation of antimicrobial resistance, which is an increasingly critical issue in food animal production [35]. Additionally, knowledge of prevalent pathogens facilitates the adoption of science-based biosecurity practices, such as pre-movement screening of asymptomatic carriers, which is essential for preventing disease spread during animal transport or herd expansion [36]. From an economic and public health perspective, this assay supports sustainable livestock production by enabling early intervention, reducing losses due to morbidity and mortality, and lowering overall treatment costs [37].

One limitation of the current study is the absence of an internal amplification control (IAC) for monitoring PCR inhibition or RNA/DNA extraction efficiency. This was primarily due to the limited number of available fluorescence channels in the commonly used real-time PCR instruments in most veterinary diagnostic laboratories. Including an IAC would require sacrificing one of the target detection channels, potentially reducing the assay’s multiplexing capability. In addition, BRDC is not caused solely by the eight pathogens included in this study. Other agents such as *Histophilus somni*, *Trueperella pyogenes*, and bovine herpesvirus 4 are also recognized as contributors to BRDC [38]. In selecting the target pathogens for this assay, we reviewed the published literature and epidemiological data from various regions, focusing on the most commonly reported four RNA and four DNA pathogens associated with BRDC [8,17,39]. Despite this targeted approach, our long-term goal remains to develop a more comprehensive, rapid, and cost-effective detection system capable of covering a broader range of BRDC-associated agents. To better meet the demands of clinical diagnostics, we plan to incorporate additional BRDC-associated pathogens as well as internal control in future assay updates, aiming to further enhance its performance and reliability.

## 5. Conclusions

In conclusion, this study successfully developed a rapid, sensitive, and cost-effective multiplex qPCR assay for the simultaneous detection of eight major pathogens associated with BRDC. The assay demonstrated high specificity, sensitivity, and reproducibility. This diagnostic tool offers significant advantages for early pathogen identification and disease monitoring, which are essential for optimizing BRDC management and improving animal health outcomes. With its potential for broad application in veterinary diagnostics, the assay contributes to enhanced diagnostic efficiency, reduced economic impact, and more responsible use of antimicrobial agents in cattle production systems.

## 6. Patents

A patent application has been submitted for this research, with the following numbers: application (202510313030.6), online public examination (CN119876495A).

## Figures and Tables

**Table 1:** Sequences of primers and probes of eight BRDC pathogens.

Primer/Probe ^1^	Target Gene	Sequences (5′–3′)	Reference Strain Genbank No.
BVDV-F	5′ UTR	AGCCATGCCCTTAGTAGGACTA	PP992316.2
BVDV-R		GTCCACGTGGCATCTCGA	
BVDV-P		FAM-CGACTACCCTGTACTCAG-MGB	
BPIV3-F	N	TTTAGACAAGACGGGACAGTC	HQ530153.1
BPIV3-R		TGCTGAGACCTCATGATCGCT	
BPIV3-P		VIC-AATCCAGTCTGGTATTAAGTG-MGB	
BRSV-F	N	AAGGAATCTTTGCAGGGTTATTC	MN316653.1
BRSV-R		CTGGCATGACCAAGCATAATGT	
BRSV-P		ROX-TGAATGCATATGGAGCAGGT-MGB	
BcoV-F	N	ACCCAAGTAGCGATGAGGC	MK903505.1
BcoV-R		AGGAGCAGACCTTCCTGAGC	
BcoV-P		Cy5-TGGCACGGTACTCCCTCAG-BHQ2	
M.bovis-F	oppD	CTTTGCCTTAGAAATTGACTATGAA	CP045797.1
M.bovis-R		GTCTTTGTATTTTTGGTGCATCAG	
M.bovis-P		FAM-AGTCATCATAAAGCAGCAACG-MGB	
PM-F	kmt1	GACATTACTGCTCTATCCGCTATT	CP003313.1
PM-R		CCCACTCACAACGAGCCATA	
PM-P		VIC-CCATTTCCCATTTCAAGTGG-MGB	
MH-F	lktA	CGACCGAGTTCACTATAGCCGT	JQ423930.1
MH-R		CGATTTACGGTATAACTACCTTGCTC	
MH-P		ROX-CTTTAACTATTGATGCAACCA-MGB	
IBRV-F	gB	GGCGGACGAAATGCTGCG	MK654723.1
IBRV-R		GTGTGGCTGTCGCTCACA	
IBRV-P		Cy5-TTCCGCTTCACGGCCCGCTCGC-BHQ2	

^1^ F stands for the forward primer, R stands for the reverse primer, and P stands for the probe.

**Table 2:** Optimization of primer and probe concentrations.

Primer Pool	Reagent ^1^	Volume/Reaction
Primer Pool A	2 × Hifair^®^ Ⅲ P buffer	10 μL
	Hifair^®^ UH Ⅲ Enzymes	1 μL
	BVDV-F/BVDV-R	0.2 μL or 0.3 μL or 0.4 μL or 0.5 μL or 0.6 μL
	BVDV-P	0.1 μL or 0.2 μL or 0.3 μL or 0.4 μL or 0.5 μL
	BPIV3-F/BPIV3-R	0.2 μL or 0.3 μL or 0.4 μL or 0.5 μL or 0.6 μL
	BPIV3-P	0.1 μL or 0.2 μL or 0.3 μL or 0.4 μL or 0.5 μL
	BRSV-F/BRSV-R	0.2 μL or 0.3 μL or 0.4 μL or 0.5 μL or 0.6 μL
	BRSV-P	0.1 μL or 0.2 μL or 0.3 μL or 0.4 μL or 0.5 μL
	BcoV-F/BcoV-R	0.2 μL or 0.3 μL or 0.4 μL or 0.5 μL or 0.6 μL
	BcoV-P	0.1 μL or 0.2 μL or 0.3 μL or 0.4 μL or 0.5 μL
	RNA template	3 μL
	nucleic acid-free water	Up to 20 μL
Primer Pool B	2 × Taq Pro HS U^+^ Probe Master Mix	10 μL
	M.bovis-F/M.bovis-R	0.2 μL or 0.3 μL or 0.4 μL or 0.5 μL or 0.6 μL
	M.bovis-P	0.1 μL or 0.2 μL or 0.3 μL or 0.4 μL or 0.5 μL
	PM-F/PM-R	0.2 μL or 0.3 μL or 0.4 μL or 0.5 μL or 0.6 μL
	PM-P	0.1 μL or 0.2 μL or 0.3 μL or 0.4 μL or 0.5 μL
	MH-F/MH-R	0.2 μL or 0.3 μL or 0.4 μL or 0.5 μL or 0.6 μL
	MH-P	0.1 μL or 0.2 μL or 0.3 μL or 0.4 μL or 0.5 μL
	IBRV-F/IBRV-R	0.2 μL or 0.3 μL or 0.4 μL or 0.5 μL or 0.6 μL
	IBRV-P	0.1 μL or 0.2 μL or 0.3 μL or 0.4 μL or 0.5 μL
	plasmid template	3 μL
	nucleic acid-free water	Up to 20 μL

^1^ F/R/P represent the forward primer, the reverse primer, and the probe, respectively. The concentrations of all primers and probes were 10 μmol/L.

**Table 3:** Validation results for Primer Pools A and B.

Nucleic Acid Template	Primer Pool A				Primer Pool B			
	BVDV	BPIV3	BRSV	BcoV	M.bovis	PM	MH	IBRV
BVDV RNA	19.57 ^1^	−	−	−	−	−	−	−
BPIV3 RNA	−	20.11	−	−	−	−	−	−
BRSV RNA	−	−	19.78	−	−	−	−	−
BcoV RNA	−	−	−	20.53	−	−	−	−
M.bovis plasmid	−	−	−	−	20.79	−	−	−
PM plasmid	−	−	−	−	−	21.82	−	−
MH plasmid	−	−	−	−	−	−	20.58	−
IBRV plasmid	−	−	−	−	−	−	−	21.42
Mixture of eight Nucleic Acids ^2^	19.66	20.21	20.05	20.69	20.81	21.81	20.64	21.31

^1^ The value refers to the Ct value of the qPCR amplification curve. “−” indicates that there was no amplification curve detected for the reaction. ^2^ Mixture of eight nucleic acids were obtained by mixing four plasmids and four RNA transcripts containing the above targets including BVDV, BPIV3, BRSV, BcoV, M.bovis, PM, MH, and IBRV in equal amounts.

**Table 4:** Results of sensitivity experiment.

Primer Pool	Template	Positive Rates (%)			
		(10 Copies/μL)	(5 Copies/μL)	(1 Copy/μL)	(0.5 Copies/μL)
A	BVDV RNA	100	100	50	12.5
	BPIV3 RNA	100	100	37.5	0
	BRSV RNA	100	100	37.5	12.5
	BcoV RNA	100	100	62.5	12.5
B	M.bovis plasmid	100	100	25	12.5
	PM plasmid	100	100	25	25
	MH plasmid	100	100	37.5	12.5
	IBRV plasmid	100	100	12.5	0

**Table 5:** Results of reproducibility tests.

Primer Pool	Template	Copies of Template (Copies/μL)	Ct Value ^1^	CI ^2^	CVs (%)	
					Inter-Assay	Intra-Assay
A	BVDV RNA	10^5^	21.11 ± 0.05	[21.09, 21.13]	0.22	0.30
	BPIV3 RNA	10^5^	20.63 ± 0.07	[20.59, 20.67]	0.36	0.63
	BRSV RNA	10^5^	20.35 ± 0.07	[20.31, 20.38]	0.37	0.76
	BcoV RNA	10^5^	22.10 ± 0.04	[22.08, 22.12]	0.20	0.36
	BVDV RNA	10^2^	31.37 ± 0.10	[31.32, 31.42]	0.32	0.53
	BPIV3 RNA	10^2^	31.05 ± 0.12	[30.98, 31.11]	0.40	0.58
	BRSV RNA	10^2^	30.84 ± 0.15	[30.77, 30.91]	0.49	0.91
	BcoV RNA	10^2^	32.40 ± 0.12	[32.35, 32.46]	0.36	0.48
B	M.bovis plasmid	10^5^	22.06 ± 0.12	[22.00, 22.12]	0.54	0.94
	PM plasmid	10^5^	21.98 ± 0.14	[21.92, 22.05]	0.62	1.27
	MH plasmid	10^5^	21.21 ± 0.10	[21.15, 21.26]	0.49	0.92
	IBRV plasmid	10^5^	22.53 ± 0.08	[22.49, 22.57]	0.37	0.74
	M.bovis plasmid	10^2^	32.40 ± 0.29	[32.26, 32.55]	0.88	1.58
	PM plasmid	10^2^	32.37 ± 0.39	[32.17, 32.56]	1.19	2.00
	MH plasmid	10^2^	31.82 ± 0.33	[31.65, 31.99]	1.05	1.52
	IBRV plasmid	10^2^	32.95 ± 0.32	[32.79, 33.11]	0.97	1.79

^1^ The mean Ct value and standard deviation were calculated and recorded in the table. ^2^ The 95% confidence interval has been calculated, with the number preceding the square brackets representing the lower limit of confidence and the number following it representing the upper limit of confidence.

**Table 6:** Profiles of mixed infections in clinical samples from farms A and B.

Farm	Co-Infection Level	Co-Infected Pathogens (Details) ^1^	Counts
A	Single infection	BcoV	31
		BVDV	28
		PM	19
		MH	10
	Dual infection	BcoV + PM	33
		BVDV + PM	24
		BVDV + BcoV	22
		PM + MH	22
	Triple infection	BcoV + PM + MH	18
		BVDV + BcoV + PM	9
		BVDV + BcoV + MH	6
		BVDV + PM + MH	4
	Quadruple infection	BVDV + BcoV + PM + MH	16
B	Single infection	MH	59
		IBRV	6
		BcoV	1
		PM	1
	Dual infection	IBRV + MH	15
		BcoV + PM	4
		BcoV + IBRV	4
	Triple infection	BcoV + IBRV + PM	2
		BcoV + IBRV + MH	1

^1^ Abbreviations used for pathogens in this table are defined as follows: BVDV = bovine viral diarrhea virus, BcoV = Bovine Coronavirus, PM = Pasteurella multocida, MH = Mannheimia haemolytica, and IBRV = infectious bovine rhinotracheitis virus.

**Table 7:** Comparison of the developed multiplex qPCR assay and reference methods.

Reference Method No.	Pathogen	Kappa Test	Developed Method		Total	Sensitivity (%)	Specificity (%)	Agreement Rate (%)
			**+**	**−**				
[24]	BVDV	+	103	4	107	94.50	96.00	95.22
		−	6	96	102			
		Total	109	100	209			
[25]	BcoV	+	132	3	135	89.79	97.00	92.71
		−	15	97	112			
		Total	147	100	247			
[26]	PM	+	148	1	149	97.37	99.00	98.02
		−	4	99	103			
		Total	152	100	252			
[25]	MH	+	145	3	148	96.03	97.00	96.41
		−	6	97	103			
		Total	151	100	251			
[27]	IBRV	+	27	0	27	96.43	100	99.22
		−	1	100	101			
		Total	28	100	128			

## Data Availability

The original contributions presented in this study are included in the article. Further inquiries can be directed to the corresponding author.

## Abbreviations

The following abbreviations are used in this manuscript:

BRDC	Bovine respiratory disease complex
BVDV	Bovine viral diarrhea virus
BPIV3	Bovine parainfluenza virus type 3
BRSV	Bovine respiratory syncytial virus
BcoV	Bovine coronavirus
M.bovis	Mycoplasma bovis
PM	Pasteurella multocida
MH	Mannheimia haemolytica
IBRV	Infectious bovine rhinotracheitis virus
qPCR	Quantitative polymerase chain reaction
LOD	Limit of detection
CV	Coefficient of variation
